# A case report of nifedipine-induced hepatitis with jaundice

**DOI:** 10.1186/s13104-018-3322-9

**Published:** 2018-04-03

**Authors:** Dimas Yusuf, Joanna Christy, David Owen, Meghan Ho, David Li, Martin J. Fishman

**Affiliations:** 1 Department of Medicine, Faculty of Medicine, University of British Columbia, Vancouver, BC Canada; 2 Beedie School of Business, Simon Fraser University, Vancouver, BC Canada; 3 Department of Pathology, Faculty of Medicine, University of British Columbia, Vancouver, BC Canada; 4 Department of Family Practice, Faculty of Medicine, University of British Columbia, Vancouver, BC Canada

**Keywords:** Nifedipine, Calcium channel blocker, Side effect, Drug-induced hepatitis, Drug-induced liver injury, DILI, Jaundice, Hepatocellular, Liver biopsy, Adverse events

## Abstract

**Background:**

Nifedipine is a generic, well-known and commonly-prescribed dihydropyridine calcium channel blocker used in the treatment of hypertension and Prinzmetal’s angina. A known but very rare and serious adverse effect of nifedipine is clinically-apparent hepatitis which can take months to resolve.

**Case presentation:**

Here we present a case of nifedipine-induced hepatitis in a 78-year-old Caucasian female with no prior history of liver or autoimmune disease. We discuss our investigative and management approach, and present a review of prior cases. We offer an approach for patients who present with signs of acute liver injury with jaundice and high elevations in serum transaminases.

**Conclusion:**

Not much is known about nifedipine-induced hepatitis due to its rare occurrence. Its prevalence is unknown. The disease appears to afflict older men and women. It can present acutely (within days) or subacutely (within 4–8 weeks after medication start) and in an idiosyncratic manner. Chronic or latent cases have also been described, some diagnosed as late as 3 years after medication start. Common symptoms include jaundice, nausea, chills, rigors, diaphoresis, fatigue, and abdominal pain. Laboratory investigations often reveal profound elevations in AST, ALT, GGT, and conjugated bilirubin. Peripheral blood smear may demonstrate eosinophilia. Histology from liver biopsy typically demonstrates infiltration of immune cells, cholestasis, and a picture of steatohepatitis. Treatment involves immediate discontinuation of the drug with supportive care. Thus far, all published instances of nifedipine-induced hepatitis were self-limiting without mortality due to fulminant liver failure. However, this disease can take months to resolve. There is no randomized evidence for other treatments such as corticosteroids.

## Background

Nifedipine-induced hepatitis is a rare type of drug-induced liver injury (DILI), which has been a subject of increased scrutiny in the medical community over the last several years. Broadly speaking, DILI is a serious complication of medication use, with a prevalence of up to 14 out of 100,000 people [1]. It is implicated in up to 33% of patients who present with acute liver injury [2], and has been cited as the most common cause of acute liver failure—about 52% of all cases according to one study [3].

The majority of DILI—up to 75% in one prospective study—is due to acetaminophen ingestion [3]. Other notable causes include antibiotics—especially amoxicillin-clavulanate [2] and fluoroquinolones [4] —as well as medications from other classes such as anti-epileptics (Table 1). DILI is typically dose-dependent, and occasionally idiosyncratic. Dose-dependent DILI is most commonly caused by large and often supratherapeutic doses of hepatotoxic drugs, in particular acetaminophen. Conversely, idiosyncratic DILI is not dose-dependent. DILI has a broad range of clinical presentations, from subclinical (or chronic hepatitis) to fulminant liver failure requiring transplantation or even death [5]. Histologically it is a diverse phenomenon, with findings that can resemble autoimmune hepatitis, cholestasis, steatosis, fibrosis, phospholipidosis, hepatic vein thrombosis, biliary sclerosis, granulomatous hepatitis, peliosis hepatis and cirrhosis [6–10]. Rarely, DILI is also associated with hepatic neoplasms, in particular adenomas, angiosarcomas, and hepatocellular carcinomas [11, 12]. For these reasons, DILI can be thought of as a “great imitator” of liver disease and should be suspected in any patient with acute or chronic hepatitis of unclear etiology.

**Table 1:** Medications commonly implicated in drug-induced liver injuries

Type	Drug class	Common medications
Dose-dependent	Aniline analgesic	*Acetaminophen*—very common, ≥ 50% of all cases of DILI [3]
	Inhaled anesthetics	Halothane
Idiosyncratic	Antibiotics and other antimicrobials	*Amoxicillin-clavulanate*, *fluoroquinolones* (moxifloxacin, levofloxacin), minocycline, tetracycline, nitrofurantoin, trimethoprim-sulfamethoxazole [2, 4], *isoniazid* [28], erythromycin [29], clindamycin, nafcillin, dicloxacillin, ampicillin, penicillin, cephalexin, rifampin, dapsone, ketoconazole, terbinafine [30], antiretrovirals [31] (efavirenz, nevirapine [9], zidovudine, stavudine [32], didanosine)
	Non-steroidal anti-inflammatories (NSAIDs)	Diclofenac, naproxen, acetylsalicylic acid [33], mesalazine, clometacin, sulindac
	Antiarrhythmics	*Amiodarone* [6], quinidine, procainamide
	Statins and other lipid-lowering agents	*Atorvastatin* [34], lovastatin [35], ezetimibe [9], fenofibrate
	Antihypertensives	*Captopril* [9], diltiazem, lisinopril, nifedipine, amlodipine [17], verapamil [24], methyldopa, hydrochlorothiazide, dihydralazine
	Antiepileptics, anticonvulsants, anxiolytics	*Phenytoin*, *valproate*, bentazepam [34], diazepam, carbamazepine [9], zonisamide
	Anti-diabetic agents	Rosiglitazone, troglitazone [9], tolbutamide
	Antipsychotics and antidepressants	Chlorpromazine [36], prochlorperazine, haloperidol, amitriptyline, imipramine
	Immunosuppressants	Glucocorticoids, methotrexate, azathioprine, cyclophosphamide, mercaptopurine
	Chemotherapeutic agents	Cisplatin, oxaliplatin, 5-fluorouracil, floxuridine, tamoxifen
	Other notable agents	*Propylthiouracil*, tacrine, allopurinol, flutamide, estradiol, vitamin A, anabolic steroids, hydroxyurea, danazol, papaverine, dantrolene

These medications are commonly associated with drug-induced liver injuries. Acetaminophen, amoxicillin-clavulanate, amiodarone, atorvastatin, and captopril account for the vast majority of cases. In particular, acetaminophen, isoniazid, propylthiouracil, phenytoin, valproate, and fluoroquinolones account for the vast majority of drug-induced fulminant liver failures [4, 5]

## Case presentation

A 78-year-old Caucasian female presented to a community hospital in Canada with jaundice, scleral icterus, and mild epigastric discomfort. She reported a 4-day history of progressive fatigue with intermittent nausea and vomiting, diminished appetite, as well as loose, lightly coloured, and foul-smelling stools. She had no evidence of coagulopathy or encephalopathy. Her past medical history included essential hypertension, dyslipidemia, and chronic obstructive pulmonary disease. She had no history of liver disease, intravenous drug use, excessive alcohol consumption, recent travel, infectious symptoms, or constitutional symptoms. She had no exposure to poisonous mushrooms or other common hepatotoxic agents. Her medications were atorvastatin 20 mg daily which she had been taking for many years, a course of clarithromycin for community-acquired pneumonia completed 28 days prior to admission, and nifedipine 10 mg daily for hypertension which was started 14 days prior to admission. She did not take any alternative or complementary therapies.

Physical examination revealed a slightly distended abdomen with diffuse tenderness to palpation, particularly in the epigastric region, and jaundice with scleral icterus. There was no rebound tenderness, guarding, or hepatosplenomegaly. She did not have any other stigmata of liver disease and the rest of her physical examination was unremarkable.

Initial laboratory investigations revealed profound elevations in all liver enzymes, bilirubin, and LDH—all indicative of significant hepatocellular injury:

- ALT 1912 U/L (29× upper limit of normal or “ULN”).
- AST U/L (42× ULN).
- ALP 439 U/L (3× ULN).
- GGT 352 U/L (6× ULN).
- Conjugated bilirubin 119 μmol/L (24× ULN).
- Total bilirubin 134 μmol/L (7× ULN).
- LDH 408 U/L (1.7× ULN).

Her INR was between 1.1 and 1.3 throughout the hospitalization, PTT was 27 s, and albumin was slightly low at 31 g/L (normal > 34 g/L). Lipase was within normal range at 288 U/L, and creatine kinase was normal at 31 U/L.

Her complete blood count was within normal limits. There was a mild monocytosis of 1.4 giga/L. Her electrolytes were within normal range. Creatinine was 65 μmol/L with an estimated glomerular filtration rate (eGFR) of 76 mL/min. Her lactate, anion gap, random blood glucose, and extended electrolytes were all also within normal limits.

Other laboratory investigations ruled out many infectious, metabolic, and autoimmune causes of acute liver injury. Serologies (IgM) for Epstein–Barr virus (EBV) and cytomegalovirus (CMV) were negative. Serologies for the human immunodeficiency virus (HIV) and hepatitis A, B, C, and E viruses (e.g. hepatitis A IgM, HBsAg, Anti-HBs, anti-HBc, anti-HBc IgM, anti-HCV, HBeAg) revealed no sign of current infection. Anti-liver kidney microsome type 1 (anti-LKM1) antibody, anti-mitochondrial antibody (AMA), anti-smooth muscle antibody (ASMA), ceruloplasmin, and immunoglobulins were negative. Alpha-1 antitrypsin was also negative. The ANA titre was 1:80, a non-specific finding in the context of the patient’s advanced age.

**Fig. 1:**
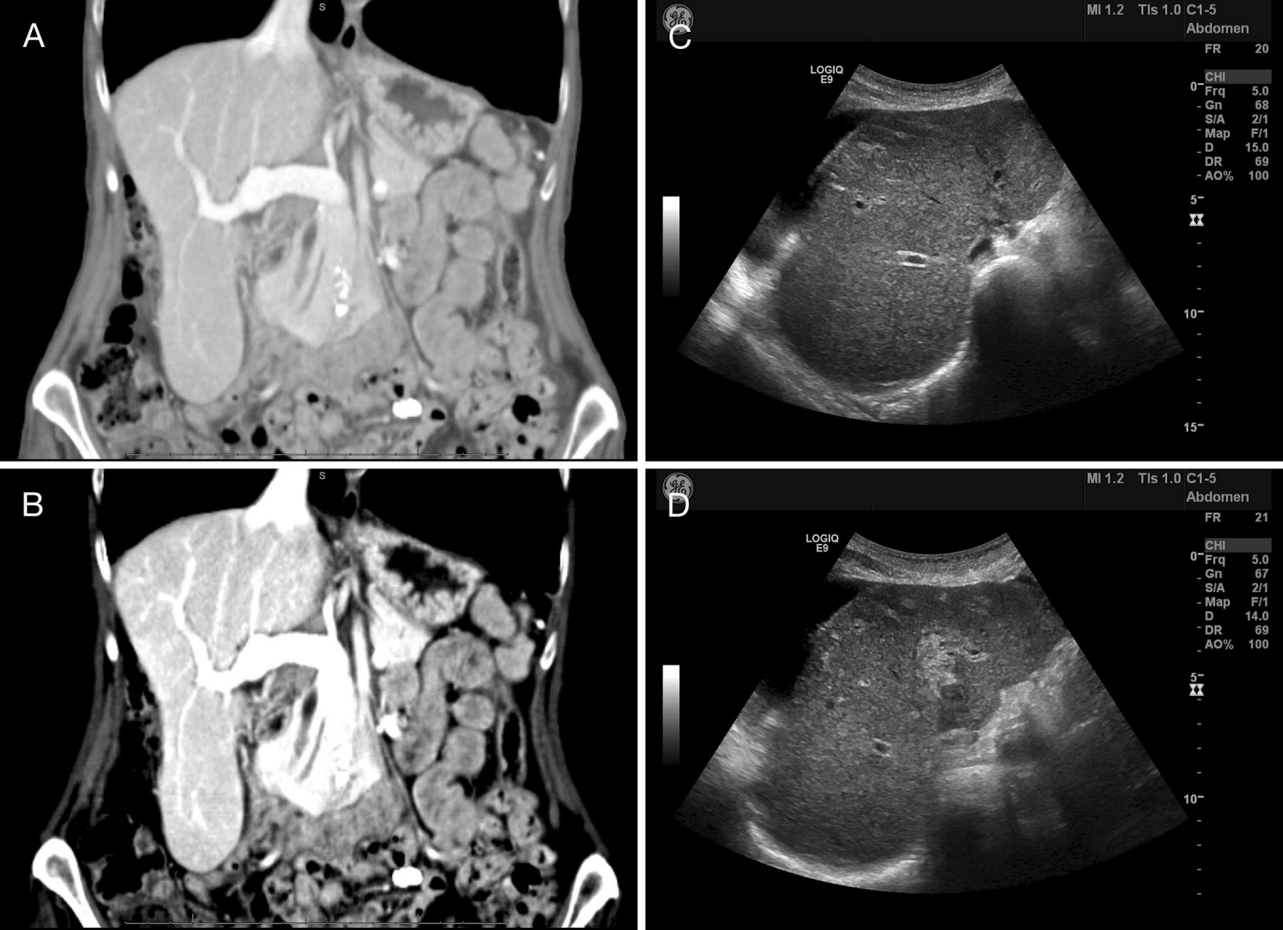
Findings on imaging. Computed tomography (CT) of the patient’s abdomen (**A**—abdominal view, and **B**—liver view) revealed a prominent Riedel lobe of the liver, with no evidence of obstruction, thrombosis, or malignancy. Mild changes of sigmoid diverticulosis were seen, with no evidence of acute diverticulitis. A repeat CT revealed mild ascites localized to the right upper quadrant, and the liver edge appeared to have a slightly nodular contour, a non-specific finding in this particular case, but which in general may suggest cirrhosis. Abdominal ultrasound (**C**, **D**) revealed a heterogeneous echotexture of the liver with regions of increased echogenicity in the right lobe, a non-specific finding which may be seen in hepatitis

A computed tomography (CT) scan of the patient’s abdomen revealed that her hepatic parenchyma was unremarkable, with no evidence of hepatic obstruction, biliary duct dilatation, thrombosis, or malignancy (Fig. 1). Incidentally, the patient was discovered to have a prominent Riedel’s lobe of the liver and sigmoid diverticulosis. A follow-up abdominal ultrasound revealed that her liver had a heterogeneous echotexture. The gallbladder was markedly thickened and edematous but decompressed.

The patient was admitted to hospital. Suspicious that her liver injury may be statin-induced, her atorvastatin was held on admission. Nifedipine was also held on admission.

Although her liver enzymes continued to steadily improve during the admission, the patient’s clinical condition deteriorated during the first 14 days of hospitalization with worsening fatigue, jaundice, mild hypotension and development of a generalized, pruritic urticarial eruption that partially responded to antihistamines. The patient then developed an acute COPD exacerbation treated with a 5-day course of prednisone, oral moxifloxacin, nebulized ipratropium and salbutamol, and supplemental oxygen.

**Fig. 2:**
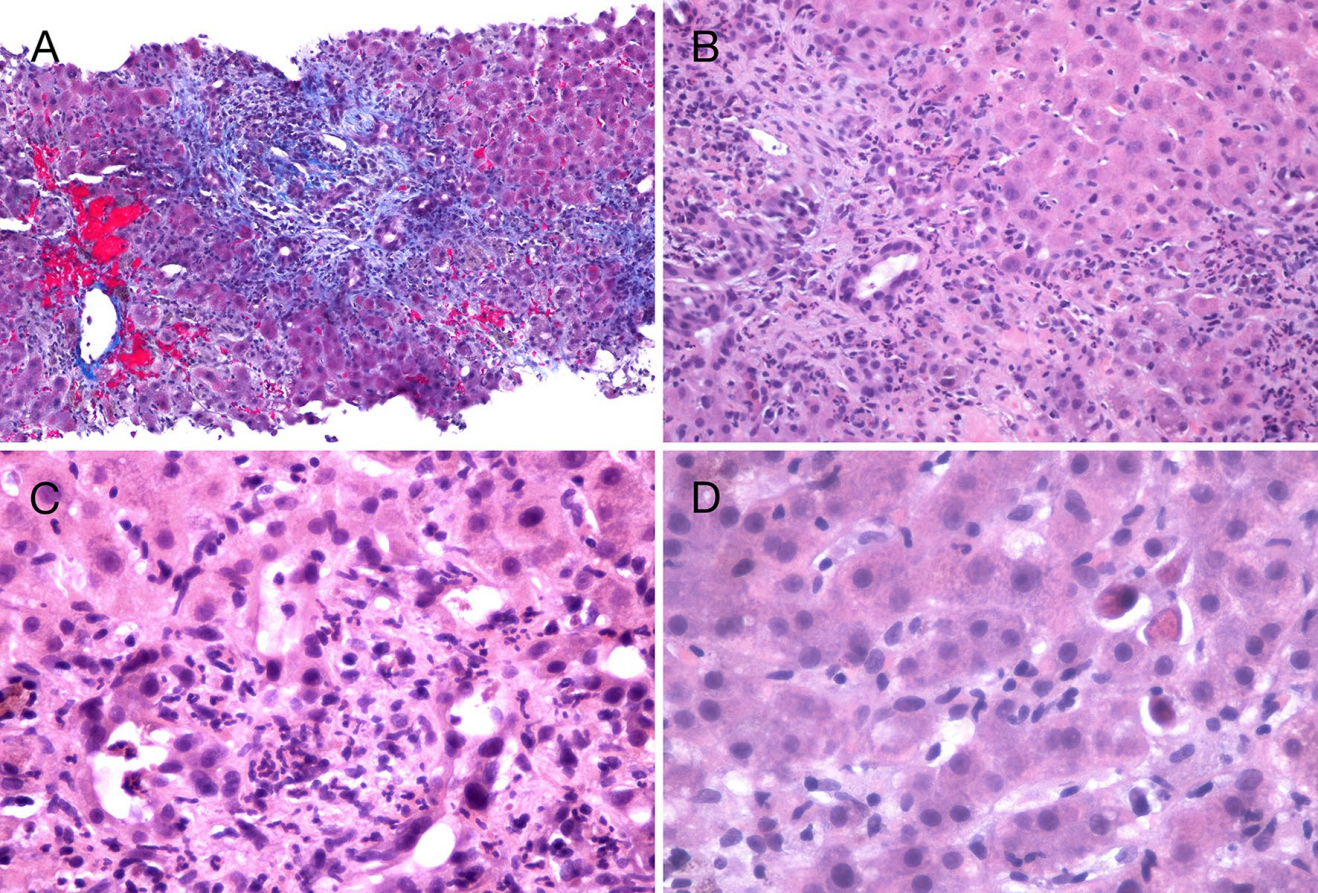
Findings on liver core biopsy. Ultrasound-guided core needle biopsy of the liver demonstrated that the overall hepatic architecture was preserved. Abnormal features included the marked expansion and fibrosis of portal tracts. In **A** fibrous tongues can be seen extending outwards into the liver parenchyma. The centrilobular areas appear preserved. There is pericentral sinusoidal congestion. Within the portal tracts (**B**) there are heavy infiltration of acute and chronic inflammatory cells, mostly lymphocytes and neutrophils. This extends into the limiting plate and extensively involves the adjacent liver parenchyma. Many damaged bile ductules and necrotic hepatocytes can be seen in **C**, and isolated necrotic hepatocytes can also be seen in the liver parenchyma in **D**. These biopsy findings are consistent with severe subacute hepatitis with developing portal fibrosis. The presence of plasma cells within the inflammatory infiltrate suggests the likelihood of an immune mechanism (hypersensitivity) as a component of the damage. However, the overall pattern of liver damage is unlike typical autoimmune hepatitis. In the absence of anti-smooth muscle antibodies, the possibility of drug-induced hepatitis via a hypersensitivity or immune mechanism is favoured. In particular, bile ductular damage—which is commonly seen in drug-induced hepatitis—may in turn produce hypersensitivity-related liver damage

Given the ongoing elevation in liver enzymes and jaundice, an ultrasound-guided core liver biopsy was performed. The biopsy demonstrated portal tract and central zone inflammation with necrosis and plasma cell infiltrates, which was consistent with a hypersensitivity-mediated drug-induced hepatitis (see Fig. 2).

Plasma cell infiltration of the liver, seen in our patient’s liver biopsy, can be a sign of autoimmune hepatitis. However, in this case, we felt that the overall histologic pattern of liver damage was not consistent with what would be typically seen in autoimmune hepatitis. This, coupled with the absence of immunological markers associated with autoimmune hepatitis, made the diagnosis of autoimmune hepatitis less likely.

In addition, the patient’s presentation is very consistent with published cases of nifedipine-induced hepatitis, which have suggested that symptoms typically manifest within 2 weeks of initial exposure. This patient’s symptoms began 10 days after she started taking the drug.

During our patient’s third week of hospital stay, her bilirubin and liver enzymes began to normalize. Her jaundice, abdominal pain, and urticaria resolved, and her energy improved. She remained in hospital for 2 additional weeks for supportive care and rehabilitation. She was discharged home after 5 weeks of hospitalization with full resolution of her liver disease and return to baseline functioning.

The limitation of our diagnosis was that it largely relied on the exclusion of other diagnoses. While it remains possible that there was another etiology, we felt the odds were small. One way to confirm the diagnosis would be to re-challenge the patient with nifedipine after she has recovered from the illness, to see if a similar reaction recurred. However, given the severity of the patient’s liver injury, we did not have the option to do this.

The patient’s other medications—in particular atorvastatin and clarithromycin—have also been associated with drug-induced hepatitis. However, for various reasons discussed below, we felt that nifedipine was the most likely cause.

The possibility that the patient’s liver injury was caused by atorvastatin was strongly considered, however ultimately, we believe this to be less likely as the patient had been taking atorvastatin for years with no complications. Twelve weeks after the patient was discharged from hospital, the patient was re-challenged with atorvastatin at gradually increasing doses and experienced no liver injury.

Clarithromycin has been known to cause liver enzyme elevations in 1–3% of patients exposed to the drug, and rarely, it can also cause fulminant hepatitis similar to nifedipine. One key difference is that a number of deaths have been reported in the literature for clarithromycin, whereas none have been reported for nifedipine [13]. The histology of both statin and clarithromycin-induced hepatitis can be similar to the histology of nifedipine-induced hepatitis. All can exhibit signs of cholestasis [14] and infiltration of immune cells [15]. Despite these similarities, the diagnosis of clarithromycin-induced hepatitis was felt to be less likely for our patient as she has been exposed to clarithromycin on multiple occasions for COPD exacerbations over the past several years, and none of her previous exposures to clarithromycin has resulted in drug-induced liver injury. Furthermore, the onset and peak of liver injury in relation to the timing of clarithromycin exposure is not typical of clarithromycin-induced liver injury—at least for the ones that have been reported. A recent review of the literature on this topic demonstrated that the vast majority of fulminant liver injuries caused by clarithromycin occur within days of administration, usually within 1 week [13]. Onset of fulminant liver injury that occurs 3 weeks after discontinuation of clarithromycin has virtually been unreported.

## Discussion and conclusions

Nifedipine is a dihydropyridine calcium channel blocker, initially developed in Germany in the 1970s [16]. It remains a commonly prescribed medication for hypertension and Prinzmetal angina. Common side effects include facial flushing (up to 25%), peripheral edema (up to 30%), dizziness or lightheadedness (up to 27%), and gastrointestinal symptoms such as nausea and heartburn (up to 11%) [17]. A very rare but known adverse effect of nifedipine is drug-induced liver injury, which has been described in the literature in nine case reports and reviews that were published between 1979 and 1999 [14, 15, 17–23] [14, 15, 17, 19–23].

### Characteristics of nifedipine-induced hepatitis

Not much is known about nifedipine-induced hepatitis due to its rare occurrence. The disease appears to afflict older men and women, with an age range of 59–80 years old as per published case reports [13, 14, 16–21] [13, 14, 17–22] [14, 15, 17–22] [14, 15, 17, 19–23].

Nifedipine-induced hepatitis is often acute and idiosyncratic, with symptoms occurring as rapidly as within 12 h to as late as 1 month after initial ingestion [14, 17]. Indolent cases have been described, diagnosed as late as 3 years after initial ingestion [14, 23]. Common presenting symptoms are jaundice, nausea, chills, rigors, diaphoresis, fatigue, and abdominal tenderness.

Biochemically, the initial pattern of liver injury is profound elevation of liver enzymes and conjugated bilirubin. Peripheral blood smear may reveal eosinophilia [15]. Data from published cases reflect an average elevation of AST of 17× ULN, ALT 7× ULN, GGT 16× ULN, and conjugated bilirubin of 14× ULN. Total bilirubin was also frequently elevated but to a lesser degree at 5× ULN, as were LDH (3× ULN) and ALP (3× ULN) (Table 2). Immunological markers of autoimmune hepatitis, ANA, ASMA, AMA, and anti-LKM1 are typically negative or inconclusive.

**Table 2:** Characteristics of nifedipine-induced liver injury from prior case reports

Case report	Rotmensch et al. [16]	Davidson [37]	Shaw et al. [14]	Babany et al. [13]	Basile et al. [21]	This case
Patient	69-year-old male treated for stable angina	59-year-old male treated for mild angina	80-year-old female treated for crescendo angina	78-year-old female treated for arterial hypertension	76-year-old male with end stage renal disease	78-year-old female treated for hypertension
Nifedipine dose	40 mg PO daily	–	60 mg PO daily divided into 3 doses	20 mg PO daily	60 mg PO daily (extended release)	10 mg PO daily
Course of illness						
Onset of symptoms after initial dose	10 days, 12 h (re-exposure)	14 days	2–5 days	1 month	3.25 years	10 days
Peak after initial dose	–	–	5 days	–	3.5 years	14 days
Peak after onset of symptoms	–	–	1–3 days	–	90 days	4 days
Resolution of clinical or biochemical signs of disease following cessation	Up to 10 months	Up to 6 months	Up to 2 months	Up to 2 weeks	Up to 3 months	Up to 5 weeks
Presenting symptoms						
Unwell or fatigued	–	Yes	–	–	–	Yes
Jaundice	Yes	Yes	–	–	Yes	Yes
Nausea	Yes	–	Yes	–	–	Yes
Anorexia	Yes	–	Yes	–	–	Yes
Chills, rigor	Yes	Yes	–	–	–	Yes
Fever	Yes (38.8 °C)	–	No	–	–	No
Diaphoresis	–	Yes	–	–	–	No
Abdominal pain	–	–	Yes, RUQ	–	–	Yes
Ascites or hepatomegaly	No	–	–	–	–	No
Biomarkers at peak						
Bilirubin, conjugated Average elevation: 14.0× ULN	8.6× ULN 3 mg/dL (ref ≤ 0.35 mg/dL)	–	–	–	9.8× ULN 3.44 mg/dL (ref ≤ 0.35 mg/dL)	23.8× ULN 119 μmol/L (ref ≤ 5 μmol/L)
Bilirubin, total Average elevation: 4.6× ULN	–	–	1.5× ULN 35 μmol/L (ref ≤ 24 μmol/L)	–	4.8× ULN 6.24 mg/dL (ref ≤ 1.3 mg/dL)	7.4× ULN 134 μmol/L (ref ≤ 18 μmol/L)
AST (SGOT) 16.8× ULN	1.12× ULN 56 U/L (ref ≤ 50 U/L)	–	21.3× ULN 1065 U/L (ref ≤ 50 U/L)	“Normal”	2.9× ULN 146 U/L (ref ≤ 50 U/L)	41.9× ULN 1592 U/L (ref ≤ 380 U/L)
ALT (SGPT) Average elevation: 6.8× ULN	1.2× ULN 68 U/L (ref ≤ 55 U/L)	0.9× ULN 49 U/L (ref ≤ 55 U/L)	–	“Normal”	1.4× ULN 79 U/L (ref ≤ 55 U/L)	29.4× ULN 1912 U/L (ref ≤ 65 U/L)
ALP Average elevation: 2.8× ULN	420 U/dL	20 KA units	3.2× ULN 352 U/L (ref ≤ 110 U/L)	1.5× ULN	3.8× ULN 955 U/L (ref ≤ 250 U/L)	2.7× ULN 439 U/L (ref ≤ 160 U/L)
GGT Average elevation: 16.4× ULN	–	31.8× ULN 1592 U/L (ref ≤ 50 U/L)	24.5× ULN 735 U/L (ref ≤ 30 U/L)	5.3× ULN	14.0× ULN 699 U/L (ref ≤ 50 U/L)	6.4× ULN 352 U/L (ref ≤ 55 U/L)
LDH Average elevation: 3.4× ULN	–	–	5.2× ULN 1190 U/ (ref ≤ 230 U/L)	–	–	1.7× ULN 408 U/L (ref ≤ 240 U/L)
Immunology and hematology						
Eosinophilia	–	–	Yes	–	–	No
IgG	22.2 g/L (elevated)	–	–	–	–	12.0 g/L (normal) (ref ≤ 15.2 g/L)
ANA pattern	–	–	–	Negative	–	1:80 (positive) Homogeneous, chromosome +ve
ASMA	Negative	–	–	Negative	–	Negative
AMA	Negative	–	–	Negative	–	Negative
Anti-LKM1	–	–	–	–	Negative	1.2 EU (negative) (ref ≤ 20 EU)
Complement	Normal	–	–	–	–	–
Imaging						
Abdominal US	–	–	Normal	–	Normal	Normal
Abdominal CT	–	–	–	–	–	Hepatitis
Liver biopsy	–	“Subacute hepatitis on background of alcoholic liver disease”	“The portal tracts were expanded with a mixed inflammatory cell infiltrate rich in eosinophils”	“Alcoholic-like liver injury, consisting of steatosis and Mallory bodies”	–	“Portal tract and central zone inflammation and necrosis with plasma cell infiltrates”

This table summarizes the findings of prior case reports of nifedipine-induced hepatitis in the literature. Jaundice, nausea, anorexia, chills, rigors, and abdominal pain appear to be the most common presenting symptoms. Biochemically, there is a pattern of general transaminitis with marked elevations in AST, GGT, and conjugated bilirubin

On liver biopsy, a multitude of findings have been described in the literature. There is often a general impression of “hypersensitivity” [20] or “subacute hepatitis” [17]. Signs of cholestasis and centrilobular necrosis have been observed [14]. Other features include an alcoholic-like picture with steatosis, hypertrophic hepatocytes and Mallory bodies [14], and an expansion of portal tracts with a mixed inflammatory infiltration of lymphocytes, histiocytes, neutrophils, and eosinophils [15]. In these latter findings, the parenchyma architecture may be preserved with mild infiltration of lymphocytes, neutrophils, and eosinophils [15]. The findings of nifedipine-induced hepatitis are similar to the findings of liver injury from verapamil, another calcium channel blocker [24].

In case reports where repeat liver biopsies were obtained following clinical and biochemical resolution of the liver injury, histological changes seen during the illness—in particular steatosis and Mallory bodies—typically resolve [14].

### Diagnosis, management, and outcome of severe drug induced liver injury

DILIs are one of the few etiologies of liver injury that can result in very high elevations of liver enzymes that exceed 1000 U/L (Table 3). The main treatment for DILI is prompt discontinuation of the offending drug. Switching patients to another drug from the same class is not entirely safe as cross-reactions frequently occur. For instance, in one case of nifedipine-induced hepatitis, the patient’s jaundice recurred after he was started on amlodipine, another dihydropyridine calcium channel blocker [23].

**Table 3:** Common causes of severe liver injury

Category	Common examples	Additional investigations
Drug-induced hepatitis	Dose-dependent Idiosyncratic	Medication history, rule out other possible causes, CBC, liver biopsy
Viral hepatitis	Hepatitis A, B, C, D, and E Epstein–Barr (EBV) virus Cytomegalovirus (CMV) Human immunodeficiency virus (HIV), HSV, Parvovirus B19	Drug and travel history, hepatitis A IgM, hepatitis B surface antigen (HBsAg), anti-HBc, anti-HBs, anti-HCV, anti-HBc-IgM, HBeAg, monotest, EBV serology, EBV DNA by PCR, CMV serology, CMV DNA by PCR, HIV antibody
Alcoholic hepatitis	Ethanol ingestion	Serum ethanol level, AST to ALT ratio (2:1 or greater may suggest ethanol injury)
Toxic hepatitis	Vinyl chloride Pyrrolizidine alkaloids (i.e. found in certain teas) Poisonous mushrooms	Diet history, vinyl chloride breath test or urine test for thiodiglycolic acid
Autoimmune hepatitis	Overlap autoimmune hepatitis Type 1 autoimmune hepatitis Type 2 autoimmune hepatitis	In general, consider: total IgG, gamma-globulin level, anti-soluble liver antigen or liver pancreas (anti-SLA/LP) antibody, liver biopsy Anti-nuclear antibody (ANA), anti-smooth muscle antibody (ASMA), anti-actin antibody (AAA), anti-dsDNA antibody, anti-soluble liver antigens (SLA) antibody, anti-neutrophil cytoplasmic antibody (ANCA), anti-mitochondrial antibody (AMA) Anti-liver kidney microsomal 1 (anti-LKM1) antibody, anti-liver cytosol antigen (anti-LC1), atypical p-ANCA (pANNA), anti-soluble liver antigens (SLA)
Ischemic hepatitis	Budd-Chiari syndrome (hepatic vein obstruction) Shock	CBC, lactate, hypercoagulopathy workup, age-appropriate malignancy workup, abdominal ultrasound, CT, or MRI

The above are causes of liver injury that can result in liver enzyme elevations that exceed 1000 U/L, or 25 times the upper limit of normal

While the majority of DILIs will resolve with prompt discontinuation of the offending drug, DILIs can worsen nonetheless and progress to liver failure that requires transplantation. Long-term sequelae of DILI include ongoing impaired liver function, progression to cirrhosis, and rarely, the development of hepatic malignancies. Mortality rates for DILI vary greatly depending on the offending drug and patient population, but has been reported as up to 60% in the published literature [4]. Fortunately, nifedipine-induced liver injury tends to have a favorable outcome, and no cases of mortality or liver transplantation have been reported in the literature [14].

Additional therapeutic options are available to clinicians in select situations. Acetaminophen toxicity can be treated with n-acetyl cysteine (NAC) [25]. Valproate-induced hepatitis can be managed with activated charcoal and emergent hemodialysis [26].

Analysis of the United States Network for Organ Sharing (UNOS) liver transplant database revealed that DILI accounted for 15% of liver transplants secondary to acute liver failure. Most (76%) of the recipients were female. Acetaminophen accounted for 49% of DILI-related liver transplants, followed by isoniazid (17.5%), propylthiouracil (9.5%), phenytoin (7.3%) and valproate (7.3%). A marker for increased severity and poorer prognosis was described by Hyman Zimmerman and recently validated by two different groups in studies involving a large number of patients with suspected DILI. Named Hy’s rule, it includes the presence of jaundice with a concomitant elevation in serum bilirubin (≥ 2× ULN) and serum transaminases, in particular ALT (≥ 3× ULN) [27].

### Closing remarks

Hepatitis is a rare adverse effect of nifedipine. It typically affects older individuals who recently began taking the medication. Presenting symptoms include jaundice, nausea, chills, rigors, diaphoresis, fatigue, and right upper quadrant abdominal pain. Laboratory investigations reveal elevations in bilirubin and liver enzymes. Autoimmune serology are not expected to be positive. Imaging is usually benign or non-specific. Liver biopsy often shows a pattern of generalized inflammation, infiltration of immune cells in hepatic structures, and cholestasis. Patients will not improve unless the offending medication is discontinued. Other calcium channel blockers should be avoided if possible, as there have been reports of cross-reactivity. Treatment is supportive, as no treatments have been formally studied. Reassuringly, the nifedipine-induced DILI appears to be self-limiting although the elevations in liver enzymes can take months to resolve. No deaths from nifedipine-induced hepatitis have been reported in the literature.

In general, drug-induced hepatitis should be suspected in all patients with acute or chronic liver injury of unclear etiology. While most cases of drug-induced hepatitis resolve with prompt discontinuation of the offending agent, some may progress to fulminant and irreversible liver failure. Markers for increased severity and poorer prognosis include the presence of jaundice and elevation in bilirubin (≥ 2× ULN) and ALT ≥ 3× ULN [27]. This case serves as a reminder to clinicians to always consider the possibility of drug-induced hepatitis in patients with liver injury of unclear etiology.

## Abbreviations

ALP: alkaline phosphatase
ALT: alanine aminotransferase or alanine transaminase
AMA: anti-mitochondrial antibody
ANA: anti-nuclear antibody
ANCA: anti-neutrophil cytoplasmic antibody
Anti-LKM1: anti-liver kidney microsomal type 1 antibody
ASMA: anti-smooth muscle antibody
AST: aspartate aminotransferase
BPM: beats per minute
CMV: cytomegalovirus
COPD: chronic obstructive pulmonary disease
CT: computed tomography
DILI: drug-induced liver injury
EBV: Epstein–Barr virus
eGFR: estimated glomerular filtration rate
g/L: grams per liter
GGT: gamma-glutamyl transpeptidase
giga: unit prefix in the metric system denoting a factor of billion
HIV: human immunodeficiency virus
IADL: instrumental activities of daily living
INR: international normalized ratio
IV: intravenous
LDH: lactate dehydrogenase
mg: milligram
mL: milliliter
mL/minute: milliliters per minute
mmol/L: millimoles per liter
MRI: magnetic resonance imaging
NAC: *N*-acetyl cysteine
NSAID: non-steroidal anti-inflammatory drug
PTT: partial thromboplastin time
U/L: units per liter
ULN: upper limit of normal
umol/L: micromole per liter
UNOS: United States Network for Organ Sharing
WBC: white blood cell
